# Metabolic Reprogramming in Gliocyte Post-cerebral Ischemia/ Reperfusion: From Pathophysiology to Therapeutic Potential

**DOI:** 10.2174/1570159X22666240131121032

**Published:** 2024-02-09

**Authors:** Lipeng Gong, Junjie Liang, Letian Xie, Zhanwei Zhang, Zhigang Mei, Wenli Zhang

**Affiliations:** 1 Key Laboratory of Hunan Province for Integrated Traditional Chinese and Western Medicine on Prevention and Treatment of Cardio-Cerebral Diseases, College of Integrated Traditional Chinese Medicine and Western Medicine, Hunan University of Chinese Medicine, Changsha, Hunan 410208, China;; 2 Department of Neurosurgery, First Affiliated Hospital of Hunan University of Traditional Chinese Medicine, Changsha, Hunan 410007, China;; 3 Third-Grade Pharmacological Laboratory on Chinese Medicine Approved by State Administration of Traditional Chinese Medicine, College of Medicine and Health Sciences, China Three Gorges University, Yichang, Hubei 443002, China;; 4 School of Pharmacy, Hunan University of Chinese Medicine, Changsha, Hunan 410208, China

**Keywords:** Ischemic stroke, cerebral ischemia/reperfusion injury, metabolic reprogramming, gliocyte, pathophysiology, oxidative stress

## Abstract

Ischemic stroke is a leading cause of disability and death worldwide. However, the clinical efficacy of recanalization therapy as a preferred option is significantly hindered by reperfusion injury. The transformation between different phenotypes of gliocytes is closely associated with cerebral ischemia/reperfusion injury (CI/RI). Moreover, gliocyte polarization induces metabolic reprogramming, which refers to the shift in gliocyte phenotype and the overall transformation of the metabolic network to compensate for energy demand and building block requirements during CI/RI caused by hypoxia, energy deficiency, and oxidative stress. Within microglia, the pro-inflammatory phenotype exhibits upregulated glycolysis, pentose phosphate pathway, fatty acid synthesis, and glutamine synthesis, whereas the anti-inflammatory phenotype demonstrates enhanced mitochondrial oxidative phosphorylation and fatty acid oxidation. Reactive astrocytes display increased glycolysis but impaired glycogenolysis and reduced glutamate uptake after CI/RI. There is mounting evidence suggesting that manipulation of energy metabolism homeostasis can induce microglial cells and astrocytes to switch from neurotoxic to neuroprotective phenotypes. A comprehensive understanding of underlying mechanisms and manipulation strategies targeting metabolic pathways could potentially enable gliocytes to be reprogrammed toward beneficial functions while opening new therapeutic avenues for CI/RI treatment. This review provides an overview of current insights into metabolic reprogramming mechanisms in microglia and astrocytes within the pathophysiological context of CI/RI, along with potential pharmacological targets. Herein, we emphasize the potential of metabolic reprogramming of gliocytes as a therapeutic target for CI/RI and aim to offer a novel perspective in the treatment of CI/RI.

## 1 INTRODUCTION

**Fig. (1):**
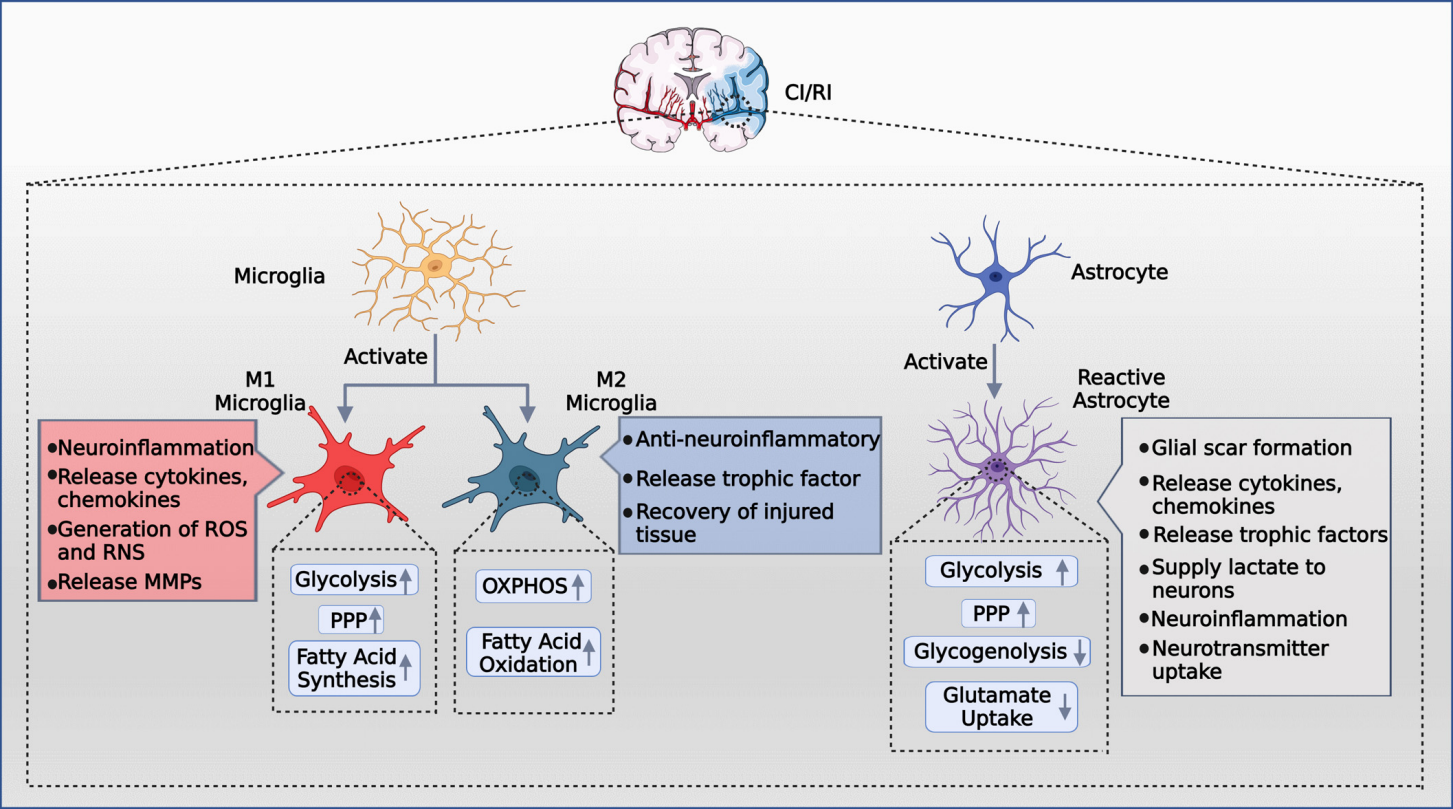
CI/RI activates microglia and astrocytes and induces metabolic reprogramming. M1 microglia increased glycolysis, PPP, fatty acid synthesis, released cytokines and, chemokines and MPPs, generated ROS and RNS, and exerted a pro-neuroinflammatory effect. M2 microglia increased OXPHOS and fatty acid oxidation, released trophic factors, repaired the damaged block, and exerted an anti-neuroinflammatory effect. Reactive astrocytes upregulate glycolysis and PPP and downregulate glycogenolysis and glutamate uptake, which may synergize with microglia to promote neuroinflammation. They also secrete trophic factors, inhibit the toxicity of excitatory amino acids and generate glial scarring to protect neurons. **Abbreviations**: CI/RI, cerebral ischemia/reperfusion injury; PPP, pentose phosphate pathway; ROS, reactive oxygen species; RNS, reactive nitrogen species; OXPHOS, oxidative phosphorylation; MMP, matrix metalloprotein. (Created with BioRender.com).

Ischemic stroke is a leading cause of disability and death worldwide [1]; however, the clinical efficacy of recanalization therapy as a preferred option is significantly hindered by reperfusion injury [2]. Recombinant tissue plasminogen activator (rtPA), an intravenous drug for treating acute is chemic stroke, is the primary approved treatment [3, 4]. However, only a small percentage of patients benefit from this treatment, and the therapeutic time window from the onset of symptoms is short [2]. Furthermore, reperfusion therapy can cause various side effects, including excitatory amino acid release, oxidative stress, calcium ion overload, inflammatory responses, and apoptosis, collectively known as reperfusion injury [2, 5]. As the existing clinical strategies for cerebral ischemia/reperfusion injury (CI/RI) are extremely limited, there is an urgent need to explore and develop novel effective drugs to treat CI/RI. Therapies targeting gliocytes are increasingly replacing neuron-centric methods, particularly microglia and astrocytes, in preclinical studies [6-8]. However, once activated, microglia and astrocytes represent a double-edged sword in the battle between neurological injury and protection, thus complicating the course and prognosis of CI/RI. Microglia and astrocytes with M2 and A2 phenotypes exhibit anti-inflammatory, neuroprotective, and regenerative activities during the acute phase of injury. In contrast, M1 and A1 interact synergistically and are involved in neuroinflammation; excessive inflammation produces neurotoxic effects [7-9]. Additionally, glial cells, including astrocytes, NG2 cells, and microglia, proliferate and become reactive gliocytes, forming a glial scar that protects the surrounding tissue from further damage. However, glial scars prevent the reconnection of neurons [10]. Microglia and astrocytes have significant therapeutic potential due to their dual protective and destructive nature, but the exact regulatory mechanism behind the phenotypic switch has not been fully investigated (Fig. **1**).

Among other effects, high rates of glycolysis and low levels of glucose oxidative metabolism due to cerebral ischemia [11], mitochondrial dysfunction, and oxidative stress due to reactive oxygen species (ROS) accumulation after reperfusion [12], causing activation of microglia and astrocytes and specific metabolic shifts in these cells [13, 14]. The expression of M2 microglia predominates in the initial phase of ischemic stroke, exerting anti-inflammatory effects, supporting tissue regeneration, and tending to utilize energy from mitochondrial oxidative phosphorylation (OXPHOS). However, later in disease progression, metabolism shifts toward lactate-driven glycolysis and significant expression of M1 microglia, which exacerbates neuroinflammation [15-17]. Besides the synergistic effect of astrocytes and microglia in neuroinflammation, metabolic coupling between astrocytes and neurons is critical for neuronal survival after ischemia [18]. Nevertheless, after CI/RI, the lactate-driven glycolytic flux of astrocytes is increased, OXPHOS activity is decreased [19], and glycogen breakdown is impaired [20], factors that may exacerbate neuronal injury. Metabolic reprogramming is an active process that can regulate stem cells, cell differentiation, and reprogramming [21]. Regulating metabolic reprogramming promises to transform microglia and astrocytes into beneficial phenotypes, making this an important area for future stroke research.

This review summarizes the current mechanisms of metabolic reprogramming in astrocytes and microglia, focusing on CI/RI. We highlight existing studies identifying potential molecular targets for metabolic reprogramming in the CI/RI treatment.

## 2 METABOLIC PATHWAYS AND REPROGRAMMING IN MICROGLIA

Metabolic reprogramming is a process by which cells upregulate various metabolic pathways to alter their phenotype, balance energy, and building-block requirements. A typical example is the Warburg effect in tumor cells, in which most cancer cells convert glucose into lactate *via* aerobic glycolysis instead of completely oxidizing it in the tricarboxylic acid (TCA) cycle [22]. Recent studies have demonstrated that activated microglia can undergo cytoskeletal changes, functional phenotypic changes, and cytokine production by reprogramming their cellular metabolism [23]. Specifically, studies have depicted that microglia prefer glucose oxidation [15] and fatty acid oxidation [24] under anti-inflammatory stimuli, while glycolysis [16] and fatty acid production [25] occur in response to proinflammatory stimuli. Recently, amino acids have also been discovered to regulate microglial functions [15]. Altering specific metabolic profiles with drugs and other agents may inhibit microglial phenotypic transformation or induce microglia to adopt favorable phenotypes. We first discuss the mechanisms of metabolic transformation of microglia discovered in various central nervous system (CNS) diseases and then discuss the metabolic reprogramming mechanisms and possible therapeutic targets in CI/RI. Despite the different etiologies of various CNS diseases, the metabolic reprogramming process of microglia is almost comparable [21], which can serve as a reference for exploring the metabolic transformation mechanisms of microglia in CI/RI.

### 2.1 Microglial Glucose Metabolism in Health and Disease

The CNS uses glucose as a primary fuel source for energy production. Microglia can metabolize glucose to fuel glycolysis and OXPHOS, as evidenced by their ability to express the required genes [26]. Following glucose uptake by microglia, pyruvate and two ATP molecules are generated *via* the glycolytic pathway, where pyruvate can be further converted to lactate or acetyl-coenzyme A (acetyl-CoA), subsequently entering the TCA cycle for further oxidation. Finally, electron transfer through the electron transfer chain terminates mitochondrial OXPHOS and generates significant ATP molecules. Existing research demonstrates that microglia rely heavily on glucose metabolism under both healthy and pathological conditions [18].

#### 2.1.1 Aerobic Glycolysis Activation in Pro-inflammatory Microglia

Infection or injury activates microglial cells to the proinflammatory phenotype, which produces proinflammatory mediators and promotes inflammation, neurotoxicity, and overexpression of glycolysis-related genes [27]. To support proinflammatory functions, microglial metabolism shifts from OXPHOS to glycolysis in the resting state [28]. First, switching metabolism to glycolysis allows microglia to produce ATP more rapidly at the expense of some efficiency [29]. Second, this reduces the amount of pyruvate entering the TCA cycle, which contributes to the production of nitric oxide (NO) and interleukin (IL)-1 [30], and the NO produced inhibits pyruvate dehydrogenase (PD), which in turn prevents pyruvate from entering the TCA cycle [31]. In addition, the mitochondrial electron transport chain can be impaired by an excess of NO, directly lowering OXPHOS levels [32]. Finally, increased glycolysis levels lead to the production of ROS and reactive nitrogen species (RNS), which also contribute to the bactericidal effect [33].

Mechanistically, microglia upregulate glucose transporter protein 1 (GLUT1) gene expression when activated, resulting in a substantial increase in glucose uptake and glycolytic metabolism in microglia [26]. Research has displayed that the major molecular mechanism regulating glycolysis in microglia is the phosphatidylinositol 3-kinase (PI3K)-AKT-mammalian target of rapamycin (mTOR)-hypoxia-inducible factor-1α (HIF-1α) axis [34-36]. The PI3K-AKT axis increases the conversion of pyruvate to lactate [37], and activated AKT can contribute to mTOR phosphorylation and ultimately upregulate HIF-1α levels, an important protein that regulates glycolysis [38, 39]. HIF-1α induces the expression of glycolysis-regulating enzymes such as hexokinase (HK) 2, phosphoglycerate kinase 1, and lactate dehydrogenase A [40]. AMP-activated protein kinase (AMPK)-mTOR-HIF-1α is also an important regulatory pathway; however, phosphorylated AMPK negatively regulates mTOR [41]. In addition, another primary regulator of glycolysis is thought to be 6-phosphofructo-2-kinase/fructose-2,6-bisphosphatase (PFKFB) 3 [42], which is responsible for phosphofructokinase (PFK)1 activation. PFK1 regulates the conversion of fructose-6-phosphate to fructose-1,6-bisphosphate and is activated by fructose-2,6-bisphosphate (F-2,6P) generated by PFKFB3 activity [43]. Reportedly, IFN-γ + lipopolysaccharide (LPS) + amyloid β-protein (Aβ) treatment of microglia leads to an increase in PFKFB3 levels that parallels the shift in glycolysis [44, 45], and IFN-γ + Aβ also increases glycolysis in microglia, accompanied by an increase in PFKFB3, HK2, and pyruvate kinase M2(PKM2) [46], while LPS+ Aβ-induced glycolysis is inhibited by the PFKFB3 inhibitor 3PO [47]. This strongly suggests that increased PFKFB3 levels may be another important mechanism of proinflammatory microglia-driven glycolysis. Toll-like receptor 4 (TLR4) is critical for microglial activation and regulation of neuroinflammation. TLR4 activation leads to gluconeogenic reprogramming of microglia and has been demonstrated to increase lactate production and inflammatory factor secretion, inhibit succinate dehydrogenase (SDH) activity, and disrupt the TCA cycle [48]. Moreover, alpha-synuclein was recently revealed to control the glycolytic reprogramming of microglia and activate their migratory ability, which is dependent on PKM2 [49]. However, the exact mechanism has not been fully elucidated and requires further investigation.

#### 2.1.2 PPP Activation and OXPHOS Reduction in Pro-inflammatory Microglia

The TCA cycle in inflammatory microglia exhibits enzymatic breakpoints in SDH and isocitrate dehydrogenase [50], which not only accumulates metabolites but also upregulates glucose flux *via* the pentose phosphate pathway (PPP) [51]. More triphosphopyridine nucleotide (NADPH) is produced *via* PPP, which provides intermediates for fatty acid and nucleotide synthesis and promotes inflammation through ribose production and amino acid synthesis [52], all of which contribute to inflammatory gene expression and cytokine release. ROS can be produced by the multicomplex enzymatic protein NADPH oxidase (NOX), which uses NADPH as a cofactor and catalyzes the production of superoxide anions [51, 53]. NOX-derived ROS plays a crucial role in regulating immunological responses, mitogen-activated protein kinase (MAPK) activation, and microglial phagocytosis [33, 54], and a recent study has demonstrated that NOX4 promotes the glycolytic process *via* ROS, thereby promoting M1 polarization of microglial cells, exacerbating the inflammatory response, and increasing the release of inflammatory factors [55]. Moreover, glucose-6-phosphate dehydrogenase (G6PD), the first enzyme and rate-limiting enzyme of PPP, can activate the downstream nuclear factor kappa-B (NF-кB) signaling pathway and cause proinflammatory polarization of microglia [56].

Reduction of OXPHOS activity in the mitochondria is also an important component of reprogramming glucose metabolism in microglia. In response to inflammatory stimuli, microglial mitochondria divide and fuse [48], leading to a reduction in the accumulation of TCA cycle products and respiratory chain electron transport by reducing the activities of succinate dehydrogenase and cytochrome c oxidase. These effects ultimately decrease the metabolic flux of OXPHOS, regulate the stability of HIF-1α, and increase cytokine secretion [57].

In conclusion, microglia increased glycolytic and PPP activity and decreased mitochondrial OXPHOS capacity in response to various inflammatory stimuli.

#### 2.1.3 Enhancement of Mitochondrial OXPHOS in Anti-inflammatory Microglia

When stimulated with anti-inflammatory stimuli, such as IL-4 and IL-13, primary cultured mouse microglia and BV2 microglia maintain high rates of oxidative glucose metabolism and a metabolic rate equivalent to that of unstimulated cells [58]. Moreover, less lactate is produced by BV2 microglia after IL-4 activation, suggesting that glycolytic metabolism is suppressed [41]. Anti-inflammatory drugs that activate microglia decrease the flux of PPP, thereby regulating cellular NADPH levels and decreasing ROS levels [59]. This suggests that anti-inflammatory microglia maintain OXPHOS rather than glycolysis for energy production [44]. Maintaining a high level of OXPHOS allows for more effective substrate consumption by microglia to obtain more ATP. However, the rate of energy production is not as fast as glycolysis, which is more beneficial for the long-term functional activities of microglia, such as exerting long-lasting neuroprotective effects and long-term wound healing processes and releasing trophic factors after injury. In these activities, microglia must maintain high membrane fluidity for complete phagocytosis and a significant amount of energy is required for these tasks [57].

Itaconate is a novel, therapeutically promising innate immunity agent found in macrophages and has recently been found to possess important anti-inflammatory properties. The expression of immune response gene 1 (IRG1) is enhanced following metabolic reprogramming of macrophage activation, which increases glycolysis and catalyzes the decarboxylation of cis-aconite to itaconate in the tricarboxylic acid cycle. However, in brain sections lacking microglia, IRG1 protein levels are significantly reduced, demonstrating the importance of microglia as a source of itaconate in the brain [60]. Itaconate significantly reduces IL-1 production and SDH activity, or it can activate nuclear factor erythroid 2-related factor 2 (Nrf2) by alkylating Kelch-like ECH-associated protein 1 (Keap1) [61, 62]. In addition, itaconate can control immunological effects and suppress the M1 polarization of microglia *via* the Nrf2/heme oxygenase-1 (HO-1) pathway [63, 64]. Interestingly, the overall effect of endogenous itaconate on microglia is anti-inflammatory, although the inhibitory effect of itaconate on SDH leads to succinate formation. Further mechanisms should be investigated.

Notably, Dickkopf (DKK) 3, a secretory glycoprotein, plays an important role in promoting cell survival by suppressing superoxide-producing enzymes and suppressing inflammation [65]. DKK3 is an emerging target in the field of stroke, and DKK3 is thought to be potentially neurotoxic in cerebral ischemia as an inhibitor of Wnt/β-catenin signaling [66]. However, DKK3 upregulation in hemorrhagic stroke [67] and neuropathic pain [68] has been reported to attenuate neuroinflammation and induce microglial polarization from M1 to M2 [68]; however, the mechanism by which DKK3 induces microglial polarization is unclear.

In conclusion, the current data suggest that anti-inflammatory microglia are more predisposed to OXPHOS, although the specific mechanism of microglial polarization toward the M2 phenotype has not been fully elucidated by previous studies.

#### 2.1.4 Inhibition of Microglial Aerobic Glycolysis as Therapeutic Approaches in CI/RI

CI/RI-induced metabolic abnormalities are characterized by the accumulation of glucose and glycolytic intermediates, as depicted by metabolomic analysis [69]. Following cerebral ischemia, the hypoxic microenvironment promotes a shift from oxidative phosphorylation to glycolysis in the microglia. This contributes to microglia exhibiting proinflammatory responses, expressing proinflammatory cytokines, and triggering bactericidal activities to adapt to the hypoxic environment. After reperfusion, oxidative phosphorylation metabolism is temporarily restored in the early phase due to the spontaneous recovery of brain function [70]. However, several mechanisms, including an increase in oxygen radicals and other harmful factors and a significant increase in the concentration of glycolytic products, impair the metabolic function of the reperfusion zone [12], which ultimately promotes proinflammatory activation of microglia and exacerbates neuroinflammation. According to our review and summary of relevant studies [71-74], early microglial glycolytic reprogramming can be suppressed to limit excessive microglial activation and, consequently, microglial proinflammatory effects. This may also effectively attenuate microglia-induced neuronal cell death and thus be neuroprotective [75], which is a beneficial strategy for alleviating CI/RI.

The hexokinase family is the first glycolytic pathway rate-limiting enzyme [76]. Li *et al*. indicated that the neuroinflammatory responses triggered by cerebral ischemia injury can be successfully reduced by targeted HK2 suppression in microglia of male rats with middle cerebral artery occlusion (MCAO) [71]. Mechanistically, HK2 overexpression causes acetyl-coenzyme A accumulation. Subsequently, HK2 can affect the upstream effectors of histone acetylation, including the activities of histone acetylases and deacetylases and, finally, the transcriptional regulation of IL-1 [77]. Moreover, CX3CL1 and its receptor CX3CR1 are crucial for the normal execution of immunological tasks by microglia under physiological and pathological conditions [78]. CX3CL1 inhibits microglia-mediated neuroinflammatory responses and has neuroprotective effects in cerebral ischemic injury [78, 79]. Specifically, CX3CL1 promotes the *in vivo* and *in vitro* development of microglia to an anti-inflammatory phenotype while switching metabolism from glycolytic to oxidative pathways by upregulating OXPHOS-related genes and downregulating glycolytic metabolism-related genes [78].

**Fig. (2):**
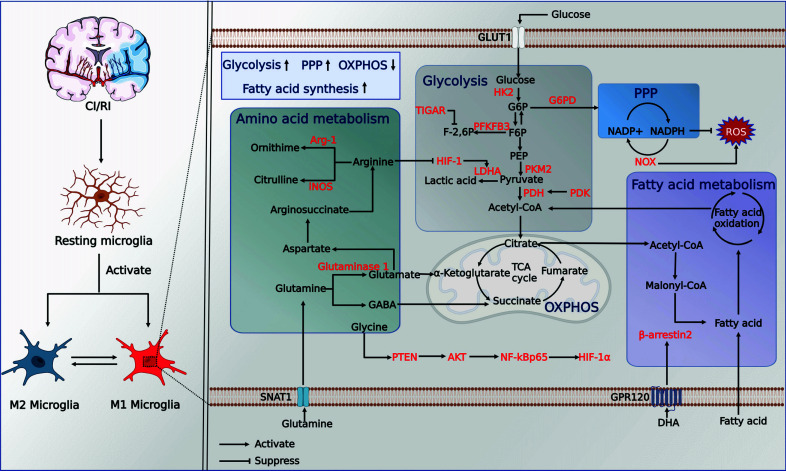
Metabolic reprogramming of microglia in CI/RI. **Abbreviations**: GLUT1, glucose transporter protein type 1; G6PD, glucose-6-phosphate dehydrogenase; NADPH, triphosphopyridine nucleotide; HK, hexokinase; G6P, glucose 6-phosphate; F6P, fructose 6-phosphate; PEP, phosphoenolpyruvate; F-2,6P, fructose 2,6-bisphosphate; PFKFB3, fructose-2,6-bisphosphatase; PKM2, pyruvate kinase type M2; PDH, pyruvate dehydrogenase; PDK, pyruvate dehydrogenase kinase; LDHA, lactate dehydrogenase A; HIF-1, hypoxia-inducible factor 1; TIGAR, TP53-induced glycolysis and apoptosis regulator; PPP, pentose phosphate pathway; G6PD, glucose-6-phosphate dehydrogenase; NOX2, NADPH oxidase 2; OXPHOS, mitochondrial oxidative phosphorylation; TCA cycle, tricarboxylic acid cycle; iNOS, inducible nitric oxide synthase;Arg-1, arginase 1; GABA, gamma-aminobutyric acid; SNAT1, sodium-coupled neutral amino acid transporter 1;GPR120, G protein-coupled receptor 120; DHA, Docosahexaenoic acid. (Created with BioRender.com).

Moreover, recent studies have displayed that chemokine-like factor 1 (CKLF1), an ischemia-induced expression protein, promotes proinflammatory activation and phagocytosis of microglia upon acute exposure by increasing their glycolytic metabolism, which is dependent on the AMPK/mTOR pathway. However, repeated administration of CKLF1 did not activate microglia, which are characterized by decreased cytokine production, phagocytosis, and glycolytic activity. This suggests that microglia acquire an immune tolerance state and that short-term blockade of CKLF1 activity improves long-term locomotor function of mice after stroke [72]. Consequently, the dual response triggered by CKLF1 in microglia may be a critical component of the inflammatory response after stroke. Dichloroacetic acid (DCA), which is known to inhibit the mitochondrial enzyme pyruvate dehydrogenase kinase (PDK), can improve oxidative glucose metabolism by increasing the pyruvate flux from the cytoplasm to mitochondria and promoting pyruvate dehydrogenase (PDH) activity [73]. In rats with acute cerebral ischemia, concomitant treatment with DAC and pyruvate reduced neuronal mortality and oxidative stress by inhibiting proinflammatory activation and improving the basal metabolic activity of microglia in the ischemic area [74]. Moreover, salvianolic acid C inhibited microglial activation of TLR4-NOD-like receptor thermal protein domain-associated protein 3 (NLRP3)-NF-кB signaling and glycolysis, preventing M1 polarization and neurotoxic effects [80]. One study found that inhibiting microglial Na/H exchanger (NHE1) increased oxidative phosphorylation, immune metabolism, and phagocytosis function, all associated with tissue remodeling and cognitive function recovery after stroke [81] (Fig. **2**).

#### 2.1.5 Improvement of Microglial PPP may Provide Neuroprotection in CI/RI

Although already highlighted in previous sections that proinflammatory microglia exhibit an increased PPP flux as one of their metabolic features, this does not necessarily mean that an increased PPP flux negatively affects the CI/RI pathological process. Post-reperfusion, microglia-mediated moderate inflammation supports the natural healing process [82], while heightened PPP flux attenuates oxidative damage to microglia, providing protection. The human oncogene TP53 is essential for controlling cellular glucose metabolism [83]. Studies have indicated that TP53-induced glycolysis and apoptosis regulator (TIGAR) improved microglial pyroptosis after CI/RI by reducing intracellular levels of F-2,6-P, decreasing PFK1 activity and flux through the major glycolytic pathway, redirecting microglial glucose metabolism to PPP, increasing production of reduced glutathione to counteract oxidative stress, and reducing reperfusion injury [34, 84, 85]. Mechanistically, F-2,6-P inhibits PPP [86], but TIGAR can act as a fructose-2,6-bisphosphatase and catalyze the conversion of F-2,6-P to fructose-6-phosphate. Consequently, TIGAR channels cellular glucose metabolism *via* PPP rather than glycolysis [84, 87].

Moreover, the PPP metabolite NADPH has antioxidant and anti-inflammatory properties [88]. It increases intracellular NADPH levels and the ratio of glutathione/oxidized glutathione after oxygen/glucose deprivation/reoxygenation (OGD/R) and decreases microglial ROS levels when exogenous NADPH is administered [34, 89]. NADPH has also been shown to protect neurons from CI/RI [89, 90]. G6PD, the key cytoprotective enzyme of PPP, has been revealed to have a neuroprotective effect during cerebral ischemia by effectively boosting neuronal PPP metabolism [91]. However, the available evidence does not support the therapeutic effectiveness of G6PD upregulation in microglia during CI/RI. Conversely, G6PD inhibition and silencing have been demonstrated to increase LPS-induced ROS production and NF-кB activation while decreasing proinflammatory activation of microglia and attenuating inflammatory responses in Parkinson's disease. This is because microglia with high G6PD activity or expression can produce excessive NADPH, which is an abundant substrate for overactivated NOX, thereby producing excessive ROS [57]. The exact functional state of microglia at different stages of the pathological process should be considered, as PPP flow in microglia under different pathological conditions does not have the same impact on pathological outcomes. However, this must be further examined and validated through further investigation.

### 2.2 Amino Acids Metabolism in Microglia

Amino acids are key immune system components and precursors of CNS neurotransmitters [92]. Succinate is a metabolite involved in the TCA cycle, and succinate accumulation in microglia during inflammation upregulates proinflammatory gene expression and mitochondrial ROS production [93, 94]. Glutamine uptake leads to conversion to glutamate by glutaminase. Subsequently, glutamate is processed by glutamate dehydrogenase to α-ketoglutarate, an essential TCA cycle metabolite. This α-ketoglutarate then enters the TCA cycle to produce succinate [95]. Alternatively, glutamate is converted to gamma-aminobutyric acid (GABA), which is then used to produce succinate [95]. Both metabolic processes increase the succinate concentration in microglia. As SDH, the enzyme that links the urea and TCA cycles, is deactivated, metabolic substrates that divert the urea cycle are also increased in proinflammatory microglia. In contrast, blocking aspartate aminotransferase, a key enzyme in the arginosuccinate shunt, reduces NO and IL-6 production [49]. Recent research suggests that amino acid metabolism, particularly glutamine metabolism [51, 96], influences the microglial phenotype.

#### 2.2.1 Reduction of Intracellular Glutamine on Microglia Promotes Neurotoxic Effects

Glutamate, a deaminated derivative of glutamine, is the major excitatory neurotransmitter in the CNS [97, 98]. *In vivo*, studies have demonstrated the metabolic flexibility of microglia [51], which, upon glucose deprivation, can take up glutamine *via* the recombinant sodium-coupled neutral amino acid transporter (SNAT) 1 and switch to glutaminolysis in an mTOR-dependent manner. This metabolic reprogramming effectively reduces the proinflammatory activation of microglia, preserves the bioenergetic function of microglial mitochondria after hypoxia and glucose deprivation [96], and attenuates microglia-mediated inflammatory responses [51]. However, interestingly, mTOR controls the transition from glucose to glutamine metabolism, while glutamine controls mTOR [99, 100].

The process by which glutamate is consumed and converted to nontoxic glutamine *via* glutamine synthetase (GS) and glutamate transporter 1(GLT-1) [22] is generally associated with astrocytes [57]; however, microglia also possess this ability [101]. Therefore, it has been suggested that microglia can eliminate extracellular excessive glutamate (converting it to glutamine) [102], which may be related to the microglial cell response to inflammatory stimuli. Reportedly, GS contributes to simple glutamine synthesis and helps regulate microglial proinflammatory activity and metabolic conversion. In a model of autoimmune encephalomyelitis, Palmieri *et al.* [103] discovered that GS expression was significantly increased in LPS-stimulated microglia, the glutamine/glutamate ratio was imbalanced when GS activity was inhibited, and glutamine homeostasis was dysregulated, resulting in a significantly increased inflammatory response. Moreover, GS inhibition may decrease the ability of active microglia to absorb glucose [103]. This finding suggests that a reduction in intracellular glutamine may promote neurotoxic effects.

#### 2.2.2 Extracellular Glutamine Increases NO Formation in Microglia

Conversely, Jayasooriya *et al.* [104] discovered that extracellular glutamine increases the expression of inducible nitric oxide synthase (iNOS) and NO formation in microglia. The phosphorylation of extracellular regulatory protein kinases (ERK) upregulates SNAT1 and SNAT2 in the presence of extracellular glutamine and LPS stimulation, thereby increasing glutamine uptake and NO formation in microglia [104]. In fact, the role of NO in the brain is highly complex. In particular, physiological amounts of NO are neuroprotective [105], and NO is essential for human physiology as an intracellular and extracellular messenger molecule [106]. However, NO synthesized and released by microglia *via* iNOS has been depicted to be an important mechanism for promoting neuroinflammation [107, 108]. Moreover, under various pathological conditions, including cerebral ischemia, large amounts of NO are produced in the brain because of induced expression of iNOS [109], and superoxide, such as ROS, can quench NO, thus quenching the bioavailability and action of NO [110]. However, the study by Jayasooriya *et al.* is not representative of real brain environments; therefore, it is unknown whether glutamine-mediated NO formation from glutamine has deleterious effects on the CNS due to the complexity of NO function.

Existing evidence suggests that intracellular and extracellular glutamine have different effects on microglia. It is important to understand how GS and related metabolic enzymes are specifically activated at different concentrations of extracellular and intracellular glutamine. This may depend on the regulation of intracellular glutamine metabolism or its relationship with different pathological conditions in the CNS [111]. Additionally, there are limited studies on glutamine metabolism in microglia under anti-inflammatory conditions, necessitating the development of more detailed metabolic mechanisms.

#### 2.2.3 Arginine and Other Amino Acids

One of the characteristic features of M2 microglia is the high expression of arginase 1 (Arg-1) [112]. iNOS and Arg-1 are enzymes that control microglial cell polarization and operation [113], and both use arginine as a substrate. The Arg-1 enzyme converts arginine to proline and polyamide, which are required for tissue repair and remodeling during wound healing [114]. In contrast, iNOS promotes M1 microglial polarization, secretion of inflammatory cytokines, and catalyzes the NO formation from arginine [115]. Studies have shown that arginine suppresses M1 microglial polarization by blocking HIF-1/LDHA signaling [116] and dampens excessive inflammatory responses [117], suggesting that arginine may have therapeutic significance as a substrate for iNOS and Arg-1 metabolism.

Glycine has neuroprotective effects in various disease models, including ischemic stroke, hypoxia, and cerebral hemorrhage [118]. Liu *et al.* demonstrated that glycine therapy reduced ischemia-induced inflammation and enhanced M2 microglial polarization [119]. Similarly, Chen *et al.* reported that homocysteine could cause brain damage *via* the Janus family tyrosine kinase 2 (JAK2)/signal transducer and activator transcription (STAT)3 pathway by activating microglia in MCAO and producing inflammatory cytokines including TNF-α and IL-6 [120]. The effect of branched-chain amino acids (BCAA) on microglia is also noteworthy, as demonstrated by Simone *et al.*, who demonstrated that high-dose BCAA treatment altered the microglial response to LPS activation [121]. Specifically, LPS-induced levels of NO, IL-1, and TNF-α were significantly reduced in the M1 phenotype, while other M2-associated genes, such as IL-10 and MRC-1, were upregulated, suggesting that the activation pattern at high BCAA levels favored the M2 phenotype. However, there was no corresponding increase in Arg-1 mRNA expression or activity, suggesting that high BCAA therapy does not cause microglia to adopt the full M2 phenotype [121].

#### 2.2.4 Glutaminase 1, a Potential Therapeutic Target for CI/RI

Disturbance of amino acid metabolism, especially the abnormally high concentrations of excitatory amino acids, is involved in the pathological process following CI/RI [122]. Glutaminase is an enzyme that deamidates glutamine hydrolytically into glutamate and ammonium ions. The well-known pathogenic effects of glutaminase are its glutamine-mediated toxic effects, first documented in 1957 [123]. Since then, glutaminase overexpression has been closely associated with acute brain disease or trauma [124]. Glutaminase 1 overexpression promotes microglial activation and exacerbates neuroinflammation and secondary brain injury in a mouse model of acute cerebral ischemia; this effect could be reduced by the glutaminase 1 inhibitor CB839. Its mechanism of action may be closely related to the release of proinflammatory exosomes [124]. This suggests that the clinical use of glutaminase 1 inhibitors is important for treating neuroinflammation. Moreover, glutaminase 1 may control microglial activation through intracellular processes. One proposed explanation is that glutaminase 1 functions as a mitochondrial enzyme that controls ROS production by regulating the ratio of two metabolites downstream of glutamate degradation, α-ketoglutarate and succinate, and that ROS activates microglia by promoting oxidative stress and activating signaling pathways such as the HIF-1α and NF-кB pathways [124].

Chen *et al.* found that arginine reduced proinflammatory markers and increased anti-inflammatory markers in microglia following CI/RI [116]. This neuroprotective effect is achieved by blocking the HIF-1α/LDHA pathway to reduce the neurotoxicity caused by excessive inflammatory responses. Liu *et al.* [119] reported that glycine suppressed NF-кB p65 and HIF-1α by downregulating phosphatase and tensin homolog deleted on chromosome 10 (PTEN) to activate the PI3K/AKT pathway.

### 2.3 Fatty Acid Metabolism in Microglia

Fatty acids are essential components of membrane lipids and an important source of energy reserves in all living organisms [125]. Fatty acids are classified into three types based on their number of double bonds: saturated fatty acids (SFAs), polyunsaturated fatty acids (PUFAs) with many double bonds, and monounsaturated fatty acids (MUFAs). When exposed to pathogenic stimuli, microglia undergo a series of changes, including cytokine release, ROS generation, chemotaxis, and pseudopod formation, which form phagocytic vesicles. All these changes require the provision of energy or biofilm expansion, which are both enabled by using lipids.

Studies have revealed that resting microglia express lipid transport proteins and several key lipid metabolism genes, including fatty acid oxidase and lipoprotein lipase (LPL) [126-130]. Transcriptional analysis of microglia isolated from Alzheimer's disease patients and mice revealed significant gene mutations related to fatty acid oxidation in microglia [131]. Single-cell sequencing revealed that microglia were significantly concentrated in areas near Aβ plaques and that apolipoprotein E (ApoE) gene expression dramatically increased when microglia were activated [132]. This evidence supports that reprogramming of fatty acid metabolism is involved in microglial activation. Moreover, when microglia are exposed to external stimuli, such as apoptotic cells or myelin remnants, lipids may act as signaling molecules to stimulate phagocytosis in microglia [130]. Therefore, fatty acids are thought to play a crucial role in the metabolism and function of microglia.

#### 2.3.1 Fatty Acid Metabolic Profiles in Microglia of Various Phenotypes

Existing research suggests that phenotypic M2 polarization can be generated in microglia and macrophages, promoting fatty acid uptake and oxidation [27] in these cells to enhance mitochondrial biosynthesis [50]. Mechanistically, microglia can express long-chain fatty acid acetyl-CoA synthase, which catalyzes the production and subsequent conversion of fatty acid acetyl-CoA *via* the β-oxidation of fatty acids that enter the TCA cycle to be used as fuel. One of the prominent features of M2 is the high expression of LPL, the rate-limiting enzyme that hydrolyzes triglyceride-rich lipoproteins and produces free fatty acids. Bruce *et al.* found that increased LPL activity was associated with higher fatty acid uptake and oxidation in M2 [24]. They discovered that LPL-deficient microglia decreased lipid uptake, while genes associated with the M2 phenotype, including Arg-1 and YM-1, were significantly downregulated in microglia. These cells also polarized to a proinflammatory phenotype characterized by iNOS, the expression of inflammatory cytokines, and a metabolic switch to glycolysis. Conversely, the alternate activation phenotype of LPL-expressing cells was maintained and included increased Arg-1 expression, insulin-like growth factor-1 (IGF-1) production, and fatty acid oxidative activity [24]. This suggests that microglia increase LPL expression to increase fatty acid oxidation and modulate M2 polarization.

Moreover, in a rat model, IFN-β administration decreased proinflammatory activation of microglia and reduced ROS and lipid peroxidation, which was associated with a shift in metabolic preference toward fatty acid oxidation [133]. L-carnitine, which helps in fatty acid transport to mitochondria, has also been shown to reverse microglia-triggered neuroinflammation [134]. It has been hypothesized that downstream activation of PPAR-γ and PPARγ-coactivator-1 (PGC-1β) induces fatty acid oxidation and mitochondrial biosynthesis, two metabolic pathways that lead to an anti-inflammatory phenotype in microglia *via* mechanisms possibly mediated by STAT6 activation [135, 136].

In contrast, proinflammatory microglia crank up genes involved in fatty acid production in response to proinflammatory stimuli, such as LPS [25]. Regarding metabolism, citric acid is first exported from the mitochondria to the cytoplasm by citric acid carriers, where it is degraded by the appropriate enzymes to acetyl-CoA, which may be a crucial building block for fatty acid synthesis [137]. Second, inflammatory stimulation increases microglial mTOR activity, which promotes cellular lipid metabolism by interacting with numerous lipid-associated proteins, including SREBP-1 and PPAR-γ [138]. Moreover, the increased NADPH induced by the high PPP flux of M1 is used as a cofactor for fatty acid formation, which ultimately promotes the expansion of the endoplasmic reticulum and Golgi apparatus to support cytokine release [139]. Finally, malonyl-CoA, an important metabolite, is increased in LPS-stimulated microglia; one of its key functions is to prevent the binding of fatty acids with carnitine in the cytoplasm by controlling carnitine acyltransferase [140]. Fatty acids that cannot bind to carnitine cannot enter the mitochondria for fatty acid oxidation, resulting in mitochondrial OXPHOS downregulation [141].

#### 2.3.2 PUFAs Induce an Anti-inflammatory Phenotype in Microglia

Phagocytosis is another important function of microglia that clears apoptotic cells, cell debris, and infections, with lipids acting as important signaling molecules [142, 143]. It has been demonstrated that PUFA can induce an anti-inflammatory or phagocyte-like phenotype in microglia [130], and lipidomic analysis [144] has depicted that oleate stimulates PUFA formation in microglia while upregulating cluster of differentiation 36 (CD36) expression. Nuclear PPAR-γ, an important switch site for anti-inflammatory responses that can be activated by fatty acids [145], can also regulate CD36 expression. Treatment with n-3 PUFAs reduced the growth of astrocytes and microglia in the striatum and substantia nigra in a mouse model of Parkinson's disease and protected the dopaminergic system in the striatum by preventing neuroinflammation and oxidative stress [146].

Mechanistically, PUFAs can inhibit lipogenesis and glycolysis by downregulating the genes involved in glucose uptake and lipid synthesis in cells. This changes the metabolic pattern of cells from glycolysis, fatty acid synthesis, and fatty acid storage to OXPHOS [147]. Docosahexaenoic acid (DHA) and eicosapentaenoic acid (EPA) can also limit the production of microglial proinflammatory mediators and enhance microglial phagocytosis by upregulating M2 and downregulating M1 signature genes such as TNF-α and IL-1 to promote M2 polarization [148]. DHA regulates inflammation within the natural range and contributes to defining neuronal function in the CNS. The brain microenvironment of DHA-deficient animals displays increased production of proinflammatory molecules, a substantial increase in the cortical microglia count, larger somas, and impaired M2 anti-inflammatory properties [149]. DHA can attenuate LPS-induced inflammation by promoting liposome production, interacting with microglial proinflammatory activity, and restoring mitochondrial function [150]. Notably, long-chain acyl-CoA synthetase 6 (Acsl6) plays an important role in DHA accumulation in the brain [151]. *In vivo* studies have demonstrated that Acsl6-deficient mice have increased microglial activation and altered glutamate metabolism, exacerbating neuroinflammatory responses in the brain, whereas Acsl6-deficient tissues have fewer DHA-containing lipids [152].

#### 2.3.3 SFAs Trigger Pro-inflammatory Responses in Microglia

Extracellular SFA has been shown to activate microglia and trigger proinflammatory responses [142, 152, 153]. Button *et al.* pretreated microglia with palmitic acid, stearic acid, and oleic acid and found that microglia secreted matrix metalloprotein (MMP)-9 activity and secretion of the proinflammatory cytokine IL-6 increased after LPS stimulation [154]. This suggests that extracellular SFA and MUFA may increase the proinflammatory invasive capacity of microglia. Interestingly, TNF-α expression in microglia significantly increased in cell cultures pretreated with MUFA, whereas it was altered negligibly after treatment with SFA [154]. Similarly, saturated fatty acids, including palmitic acid and stearic acid, can enhance microglial proinflammatory activation by activating TLR4 and NF-кB signaling pathways and increasing ROS and NO [142, 152, 153]. Moreover, following proinflammatory activation, SFA concentration in microglia often increases, consistent with the observation that SFA can trigger an inflammatory response [154].

Notably, triggering receptor 2 (TREM2), expressed on myeloid cells and microglia and functioning as an APOE receptor, is critical for regulating microglial metabolism, phagocytosis, proliferation, and survival [155]. TREM2 deficiency impairs mTOR activation, increases autophagy, and causes metabolic dysfunction in microglia [156, 157]. TREM2 also induces APOE signaling, and the APOE pathway mediates the change in microglial phenotype after phagocytosis of apoptotic neurons in mouse models of amyotrophic lateral sclerosis and Alzheimer's disease [158]. Moreover, enhancing the microglia-mediated clearance of oxidized phosphatidylcholines *via* TREM2 may help prevent neurodegeneration in MS [159]. These results suggest that TREM2 has great potential as a therapeutic target for neuroinflammation diseases.

Collectively, these data suggest that fatty acids serve as signals that mediate microglial phenotypes and functions. Generally, PUFA supports the transition of microglia to an anti-inflammatory phenotype, whereas SFA supports their proinflammatory activity.

#### 2.3.4 Omega-3 PUFA Modulation of Microglia as a Therapy for CI/RI

Compared with resting microglia, activated microglia exhibit altered concentrations of metabolites associated with fatty acid metabolic pathways after cerebral ischemia, manifested by lipid droplet accumulation and fatty acid use as fuel [160]. Additionally, adipocyte fatty acid-binding protein (A-FABP), an adipokine implicated in several metabolic diseases, is significantly overexpressed in microglia during the acute phase after cerebral ischemia [161], which exacerbates microglia-mediated blood-brain barrier (BBB) disruption through MMP-9 [154]. A recent study indicated that microglia in areas of degenerating white matter exhibit increased oxidative fatty acid metabolism activity during the chronic recovery phase following cerebral ischemia. This increase in fatty acid metabolism may be due to the breakdown of white matter debris, and itaconate may be an important regulator of this metabolic switch and the inflammatory response in degenerating white matter [162].

DHA and EPA are n-3 PUFAs that are thought to play a protective role in CI/RI [163-165]. The data also suggest that long-term supplementation of n-3 PUFA before the end of cerebral ischemia in mice may make the brain more resistant to interruptions in the blood supply. The injection of n-3 PUFA after ischemic stroke also improves brain repair and regeneration [166], cognitive and behavioral recovery after ischemic stroke [167, 168], and mice mortality [169]. Altering the lipid content of neuronal cell membranes is a possible mechanism that promotes the activity of glial cells involved in inflammatory responses and repair [170]. A recent study discovered that DHA could prevent CI/RI injury by activating the G protein-coupled receptor (GPR) 120 in two different ways: preventing microglial inflammation *via* the GPR120/β-arrestin2 pathway and preventing neuronal apoptosis *via* the PI3K/AKT pathway [171]. By reducing the release of HIF-1, iNOS, and IL-1 by astrocytes and microglia, Zendedel *et al.* [172] demonstrated that n-3 PUFA can reduce neuroinflammatory responses and exert neuroprotective activities in mice with CI/RI. Fourrier *et al.* depicted that in the hippocampal region of a mouse brain, DHA inhibited the induction of IL-6 production by LPS [173]. Specialized pro-resolving mediators (SPMs) are one of the many important bioactive substances regulating the beneficial effects of PUFA [170]. SPMs may affect tissue repair and anti-inflammation, attenuating CI/RI by reducing neuroinflammation. These effects include non-inflammatory phagocytosis promotion by microglia and macrophages. Moreover, transient local ischemia in mice causes demyelination of neurons, which damages the white matter and destroys oligodendrocytes; long-term n-3 PUFA supplementation prevents oligodendrocyte damage and reduces demyelination, which supports white matter regeneration after ischemic stroke [174].

These data suggest a beneficial therapeutic effect of n-3 PUFA in alleviating CI/RI, possibly by improving or protecting the functional status of glial cells.

## 3 METABOLIC REPROGRAMMING OF ASTROCYTES IN CI/RI

Astrocytes are critical for maintaining neuronal activity. They regulate it by stimulating synapse formation, buffering extracellular potassium ions, resisting oxidative stress, and supporting neuronal energy metabolism. Activation of astrocytes, also called reactive astrocytes, is characterized by cellular hypertrophy, proliferation, and increased glial pro-fibrillary acidic protein production within minutes of ischemic stroke onset due to inflammatory damage and ischemic causes [175]. Similar to microglia, astrocytes can exist in two very different phenotypes: A1, induced by inflammatory injury, and A2, induced by ischemia [9]. The A2 phenotype of astrocytes supports neuronal survival and tissue healing [176], whereas the A1 phenotype has neurotoxic effects [177]. Although subtypes A1 and A2 have been increasingly recognized in recent years, few of the studies we reviewed reported the use of subtypes A1 and A2 to distinguish between reactive astrocytes. Transcriptome analyses have revealed that reactive astrocytes release cytokines, chemokines, and trophic factors and exhibit proinflammatory and neuroprotective functions during acute cerebral ischemia [178, 179], demonstrating that both phenotypes of astrocytes synergize in the pathological process. Reactive astrocytes play a crucial role in neuroprotection by inducing glial scarring alongside microglia to prevent the spread of inflammation in the peri-infarct cortex and preserve brain function in the subacute phase after ischemic stroke. However, this appears to be detrimental to prolonging and repairing neurons near the lesion during the late phase [180, 181]. During the recovery phase, reactive astrocytes also promote angiogenesis, neurogenesis, synaptogenesis, and axonal remodeling [182].

Compared to microglia, given the complexity of astrocytes and the limitations of existing studies, we cannot confirm whether astrocytes are homogeneous in different brain microenvironments. Consequently, we only summarized the evidence for metabolic reprogramming in CI/RI and *in vitro* cellular experiments. Current research indicates that reactive astrocytes increase glycolytic activity while limiting mitochondrial oxidative phosphorylation in response to CI/RI. Furthermore, PPP, a glycolysis shunt channel, was also increased. This metabolic change improves the resistance to oxidative stress and ensures astrocyte survival under ischemic conditions. It also generates lactate to maintain the neuronal metabolism. However, increased glycolysis has also been associated with neurotoxic effects in reactive astrocytes after reperfusion [19]. Glycogen metabolism in astrocytes is a puzzling phenomenon. While previous research has shown that glycogen utilization should be compensatorily increased after ischemia, it has recently been demonstrated that this process is impaired after reperfusion, which may exacerbate the reperfusion injury. Additionally, glutamate metabolism significantly changes after ischemia, decreasing astrocyte-dependent glutamate-buffering capacity. Therefore, in the following sections, we will focus on the glucose, glycogen, and glutamate metabolism changes in astrocytes resulting from CI/RI and summarize some current therapeutic approaches.

### 3.1 Neuron-astrocyte Metabolic Coupling

Astrocytes are primary glycolytic cells in the CNS [183]. In astrocytes, abundant PFKFB3 activates PFK to enhance glycolysis *via* F-2,6-P transformation, while abundant PKM2 constantly upregulates glycolytic flux. PDK4, which is highly expressed in astrocytes, can inactivate PDH, preventing pyruvate from entering the TCA and ultimately allowing glucose metabolism to generate lactate mainly through glycolysis [184]. Moreover, astrocytes are the primary cells that store and use glycogen [185]. The CNS has a high energy demand because neurons constantly consume energy to maintain their action potentials and synaptic functions. Astrocytes can transport lactate produced by glycolysis or glycogenolysis out of the cell *via* the monocarboxylate transporter (MCT) 1 or 4 at the cell membrane, which is then used by neurons *via* MCT2. Therefore, this metabolic interaction considered the astrocyte-neuron lactate shuttle theory [186]. Although this theory is controversial [187], it is generally considered to explain the metabolic link between astrocytes and neurons. Following cerebral ischemia, astrocytes undergo metabolic reprogramming. First, glycogen mobilization is activated to produce sufficient glucose [188], then glycolytic metabolism is increased to ensure cell survival. Simultaneously, mitochondrial metabolism of oxidative phosphorylation is decreased [189]. A substantial amount of the generated lactate is then transported to neurons to fuel and facilitate the maintenance of synaptic connections. During prolonged cerebral ischemia, excessive accumulation of lactate leads to lactic acidosis, which alters the intracerebral milieu and damages neurons *via* various pathophysiological pathways [190-192]. Additionally, acidosis inhibits glycolysis in astrocytes, which may be one of the possible causes of energy deficiency after the onset of cerebral ischemia.

#### 3.1.1 STAT3, a Key Factor in Astrocyte Glucose Metabolic Transition

Although astrocytes upregulate glycolytic activity after cerebral ischemia has been observed, the exact metabolic changes in astrocytes are still largely unknown. Previous studies have demonstrated that STAT3 overexpression in reactive astrocytes after ischemia is closely linked to neurotoxic effects [179]. A recent study by Borbor *et al.* demonstrated that activated STAT3 triggers metabolic reprogramming of lactate-directed glycolysis in reactive astrocytes after the onset of CI/RI by initiating signaling cascades and synergistic effects with PKM2 and HIF [19]. They discovered that the OGD/R activation of STAT3 altered the transcriptional profile of astrocytes and increased the expression of Slc16a3 genes encoding LDH and MCT4 while decreasing the expression of Hk2 and Glul genes encoding HK2 and GS. Consequently, STAT3 activation in astrocytes increased glycolysis and ROS production, impaired astrocyte mitochondrial function, induced mitochondrial respiratory failure in neurons, and triggered glutamatergic synaptic loss, leading to secondary neurodegeneration [19]. Furthermore, under low blood glucose levels, astrocytes can obtain energy from stored glycogen to maintain mitochondrial respiration and oxidoreductase activity [189]. However, STAT3 activation reduces compensatory glycogen use in astrocytes. The rescue effect of STAT3 inhibition is reversed if it suppresses the glycogen consumption by astrocytes [19]. Therefore, STAT3 signaling and glycogen consumption may represent novel targets to support post-stroke recovery and play a critical role in reactive astrocytic lesions.

Interestingly, another study indicated [193] that after reperfusion, astrocytes can increase PPP metabolism by enhancing glycogen mobilization, which promotes NADPH and glutathione synthesis and ultimately decreases ROS formation. The decreased ROS levels lead to the inhibition of NF-кB and activation of STAT3, both of which cause A2-like polarization of astrocytes, leading to neuroprotection after CI/RI [193].

**Fig. (3):**
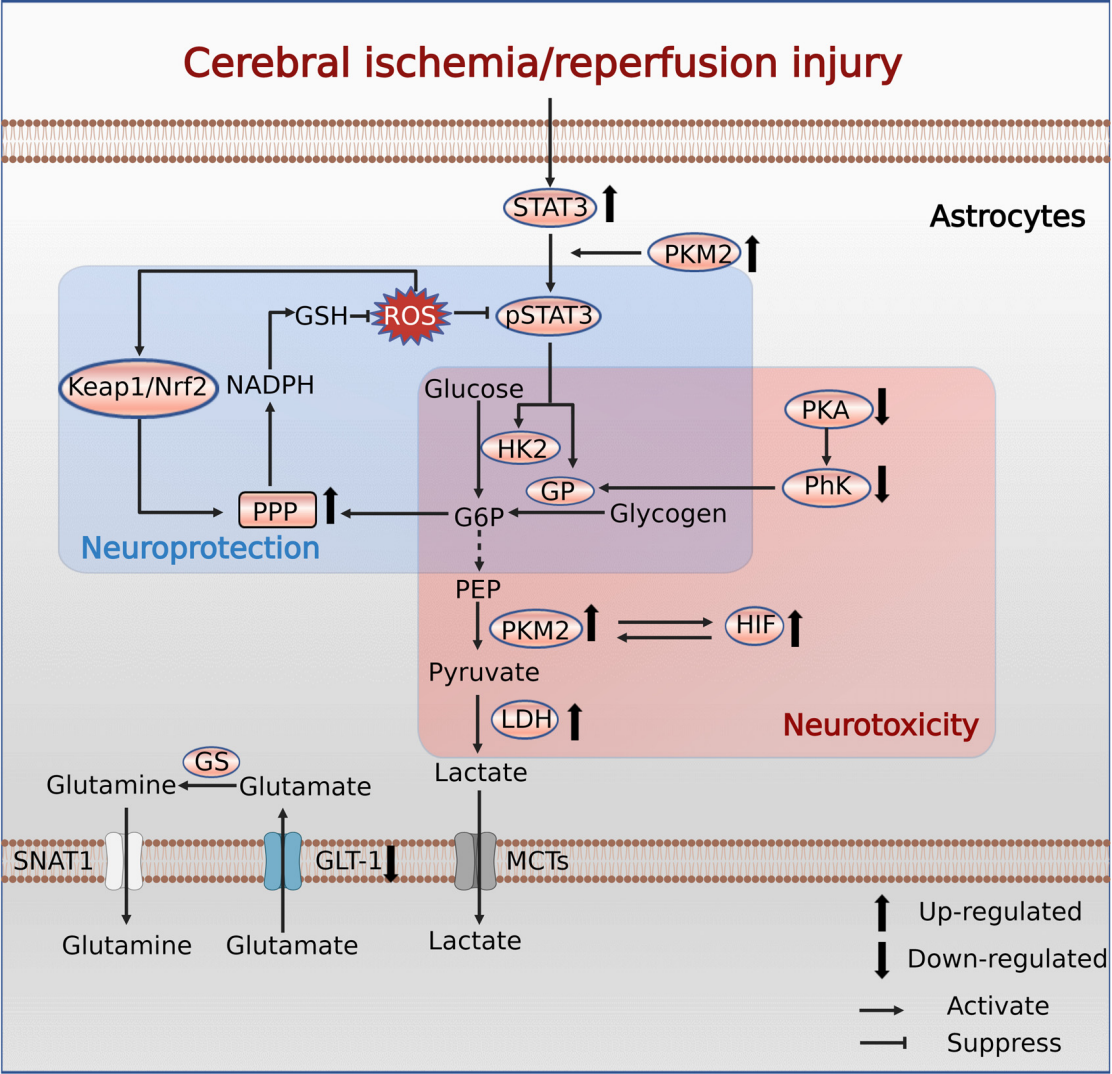
Metabolic reprogramming of astrocytes in CI/RI. **Abbreviations**: STAT3, signal transducer and activator of transcription 3; PKM2, pyruvate kinase type M2; HK2, hexokinase 2; GP, glycogen phosphorylase; G6P, glucose 6-phosphate; PEP, phosphoenolpyruvate; PhK, phosphorylase kinase; PKA, protein kinase A; HIF, hypoxia-inducible factor; LDH, lactate dehydrogenase; PPP, pentose phosphate pathway; NADPH, triphosphopyridine nucleotide; GSH, reduced glutathione; Keap1, Kelch-like ECH-associated protein 1; Nrf2, nuclear factor erythroid 2-related factor 2; GS, glutamine synthetase; SNAT1, sodium-coupled neutral amino acid transporter 1; GLT-1, glutamate transporter 1; MCT, monocarboxylate transporter. (Created with BioRender.com).

This evidence highlights the importance of astrocytic glycogen mobilization during CI/RI, and we speculate that whether astrocyte STAT3 activation is beneficial or detrimental to neurons may depend on the injury stage. Currently, STAT3 stimulates PPP and glycolytic activity during the acute phase of CI/RI, which reduces neuroinflammation and contributes to a neuroprotective effect. In contrast, glycolytic activity promoted by STAT3 during the subsequent pathological process leads to excessive proliferation of reactive astrocytes and neurotoxic effects that hinder brain healing. This is also consistent with previous studies [194], but the STAT3 mechanism of action requires further clarification (Fig. **3**).

#### 3.1.2 Impaired Glycogen Utilization in Reperfusion Injury

Glycogen is the primary endogenous energy source for the brain during cerebrovascular blockade [195]. Astrocytes can reduce brain damage from hypoglycemia during ischemia by converting reserve glycogen to glucose. Astrocytes balance gluconeogenesis and glycogenolysis to maintain the dynamic balance of glycogen [196]. Protein kinase A (PKA) and glycogen synthase kinase 3 (GSK3) control the activity of glycogen synthase, the rate-limiting enzyme for glycogen synthesis [197]. Conversely, glycogen phosphorylase (GP), the key enzyme in glycogen degradation, is controlled by the PKA glycogen phosphorylase kinase (PhK) cascade [197]. GSK3 increases in the brain with the onset of CI/RI [198], suggesting that glycogen synthase activity is impaired and accessible glycogen is limited [199].

To avoid energy shortages and minimize ischemic damage, previous studies on ischemic disease predominantly emphasized the augmentation of cellular glycogen reserves prior to the onset of ischemia [200]. A recent study offers a different perspective, showing that inhibiting PKA and PhK activity during reperfusion decreases GP activity in astrocytes, disrupting the balance between GP and glycogen synthase, which impairs glycogenolysis and ultimately leads to glycogen accumulation and worsening of reperfusion injury [20].

Promoting glycogenolysis in astrocytes may be a more promising therapeutic strategy than simply increasing intracellular glycogen reserves pre-ischemia, as shown by the restoration of the astrocytic PKA-PhK-GP cascade by insulin to maintain glycogen metabolism balance after reperfusion [20] or the use of salvianolic acid B to increase GP activity to improve astrocytic glycogen utilization after reperfusion [201]. Notably, astrocytic glycogenolytic metabolism requires careful consideration of the precise timing of ischemia and the specific pathological process; however, further research is necessary to augment this strategy effectively.

#### 3.1.3 Reperfusion-induced Activity of PPP in Astrocytes

When reperfusion begins, one of the greatest challenges is the effective removal of large amounts of ROS [202, 203]. Because astrocytes can absorb more oxygen from arterial blood than glucose, cerebrovascular occlusion-induced cerebral ischemia can be considered an ischemic pattern with a relatively adequate glucose supply [204], allowing them to increase glycolysis during hypoxic cerebral ischemia. Increased glycolytic activity and metabolic coordination [204] activate the rate-limiting enzyme G6PD in PPP [205], causing a substantial increase in PPP as a shunt mechanism for glycolysis in astrocytes after hypoxia [206]. Since NADPH produced by PPP can be used to convert oxidized to reduced glutathione (GSH), an important antioxidant that cooperates with glutathione peroxidase enzymes to destroy ROS, PPP flux is considered a key indicator of cellular antioxidant capacity [207]. Significantly, the PPP flux of astrocytes in the brain is much higher than that of neurons, suggesting that they primarily maintain the antioxidant capacity of the CNS [208]. This finding also suggests that controlling astrocyte PPP flux after reperfusion has great therapeutic potential. Few studies have indicated that astrocytes express high levels of TIGAR protein after CI/RI, and TIGAR has been shown to upregulate PPP flux in astrocytes during CI/RI and reduce NF-кB activity, thereby attenuating neuroinflammation [209], making TIGAR a potentially effective target for treating reperfusion injury.

Notably, not only does the level of glycolytic metabolism affect PPP flux, but ROS may also act as transcriptional activators of PPP. G6PD, a key enzyme in PPP, is regulated both metabolically and transcriptionally, with the Keap1/Nrf2 system emerging as a pivotal transcriptional pathway [205, 208]. Under healthy conditions, Nrf2 serves as a transcription factor that binds to the bridging protein, Keap1, to form a heterodimer. However, the ubiquitin-proteasome system continuously disrupts the Keap1/Nrf2 complex, rendering it incapable of transcription. When reperfusion occurs, massive ROS attack on Keap1 leads to Nrf2 release and translocation into the nucleus, where it binds to the appropriate transcriptional precursor and initiates target gene transcription. Thus, enhancers of the Keap1/Nrf2 system may have neuroprotective effects by increasing PPP levels in astrocytes [210-212]. Dimethyl fumarate, an Nrf2 activator, has been evaluated to reduce the proinflammatory effects of astrocytes following cerebral ischemia [213] and has shown neuroprotective effects [214].

In addition to the oxidative stress induced by high ROS concentrations, another important pathogenic mechanism of CI/RI is amino acid toxicity mediated by excitatory amino acids. Astrocytes may uptake additional glutamate that neurons release into the synaptic cleft to reduce glutamate-induced excitotoxicity. Surprisingly, a substantial PPP flux increase was observed when PPP flux was examined after glutamate delivery to astrocytes. This suggests that glutamate activates the antioxidant defense system of astrocytes [215]. Moreover, astrocyte glutamate uptake can be chemically degraded to lactate, delivered to neurons [208], or directly converted to α-ketoglutarate and used as a TCA cycle substrate [215]. This evidence suggests that glutamate uptake by astrocytes and the metabolic mechanisms involved are of potential therapeutic value in CI/RI. Therefore, in the following sections, we focus on the mechanisms of glutamate metabolism in astrocytes.

### 3.2 Astrocytes and Glutamate Toxicity

Regulating neurotransmitter homeostasis is one of the most important functions of astrocytes in the CNS, as they take up neurotransmitters released from synapses, including glutamate, GABA, and glycine, metabolize them, and release their precursors back to the neuron [216]. Glutamate is the most abundant excitatory neurotransmitter in the CNS [217]; however, excessive glutamate in the synaptic and extra-synaptic gaps leads to neuronal hyperexcitability and eventual death, a process known as glutamate excitotoxicity, which is one of the critical pathological aspects of CI/RI [218]. Therefore, swift removal of unused synaptic glutamate from the extracellular space is essential. This glutamate clearance task is primarily performed by astrocytes and is mediated by glutamate uptake transporter proteins [219]. Shortly after ischemia, the astrocyte-dependent glutamate buffering system was altered in several ways, including epigenetic regulation of GLT-1 and GLAST promoters, leading to decreased gene expression [220] and S-nitrosylation of GLT-1 with a subsequent decrease in its activity [221]. Previous studies have focused on enhancing astrocyte glutamate uptake and consumption to protect patients from glutamate toxicity in ischemic stroke.

#### 3.2.1 EAAT, an Essential Mechanism for Glutamate Uptake

Glutamate uptake is one of the most energy-intensive activities in the CNS [222]. Interestingly, astrocytes can produce more ATPs through the oxidative metabolism of glutamate than ATP consumed by glutamate uptake [223]. Most extracellular glutamate uptake occurs *via* excitatory amino acid transport proteins (EAAT) [224], which are classified as either Na+-dependent or Na+-independent transport proteins for glutamate uptake [225]. There are currently five known isoforms of EAAT [226] in mice; the first two, EAAT-1 and EAAT-2, are glutamate aspartate transporter protein (GLAST) [227] and GLT-1 [228], respectively. Except for the fraction bound by postsynaptic neuronal receptors, most synaptically produced glutamate diffuses out of the synaptic cleft and is subsequently removed from the extracellular space by EAAT-1 and EAAT-2 of astrocytes in the synaptic system [229]. Astrocytes can convert consumed glutamate to glutamine through GS, releasing it *via* SNAT1 into the extracellular milieu [230, 231] or neuronal uptake, enabling glutamate or GABA resynthesis. Alternatively, some glutamate is converted into α-ketoglutarate, a TCA cycle substrate [232]. Extracellular glutamate concentration critically influences the propensity of astrocytes to convert glutamate to glutamine or engage in oxidative metabolism [219]. Extracellular glutamate concentrations above 0.2 mM promote astrocyte oxidative metabolism, generating the energy required for glutamate uptake. Glutamate concentrations below 0.2 mM are converted into glutamine and become available to neurons [233].

Cerebral ischemia causes abnormal changes in astrocyte-dependent glutamate buffering systems. These abnormal changes include decreased epigenetic regulation of GLT-1 and GLAST promoters, leading to decreased gene expression [219], and S-nitrosylation of GLT-1, leading to a significant decrease in GLT-1 activity [221]. In the late stages of ischemia, the glutamate transporter is reversed in astrocytes due to a severe lack of energy, which results in the development of glutamate excitability and significantly affects neuron survival. Therefore, a potential treatment strategy for ischemia-reperfusion injury is to selectively increase the activity of amino acid transporter proteins in astrocytes during ischemia to promote glutamate uptake and metabolism and ultimately reduce excitotoxicity.

#### 3.2.2 GLT-1 Expression as a Credible CI/RI Mitigation Strategy

Increased glutamate intake could promote astrocyte metabolism related to the glutamate pathway, reducing the toxicity of excitatory amino acids generated during CI/RI. Several clinical trials have demonstrated that this has therapeutic potential for treating CI/RI. Based on existing data, the two main treatment approaches are protecting GLT-1 in astrocytes and promoting GLT-1 overexpression. Carnosine, a naturally occurring dipeptide, is neuroprotective by protecting GLT-1 expression in astrocytes, which lowers brain glutamate levels and reduces excitotoxicity while improving neurological function [234]. Additionally, two neuroprotective proteins, heat shock protein 72 (HSP72) and mitochondrial superoxide dismutase 2 (SOD2), have been demonstrated to reduce neuronal damage during ischemia in the forebrain by protecting astrocytes from GLT-1 and reducing oxidative stress [234]. Ceftriaxone, a GLT-1 transporter activator, promotes GLT-1 overexpression [235] and is neuroprotective in ischemic models [236, 237]. Tamoxifen, a selective estrogen receptor modulator, also increases GLT-1 expression in rat astrocytes [238] and reduces the severity of injury after reversible [239] or irreversible MCAO in rodents [240].

### 3.3 Fatty Acid Metabolism in Astrocytes

Although most energy in the CNS is derived from glucose catabolism, fatty acid oxidation contributes to approximately 20% of the oxidative energy production in the brain. Recent research suggests that astrocytes may be the main contributors to fatty acid oxidation in the brain [241].

Generally, during ischemia, the main purpose of fatty acid utilization by astrocytes is to provide fuel for β-oxidation. Thyroid hormones have been reported to counteract damage induced by ischemic stroke, and this effect depends on increased oxidative fatty acid metabolism by reactive astrocytes [242]. These reactive astrocytes with upregulated lipid metabolism demonstrated a molecular phenotype that could be helpful or protective following ischemic stroke because they shared a common gene expression profile with neuroprotective A2 reactive astrocytes [9, 178]. This suggests that astrocyte fatty acid metabolism may be involved in developing ischemic stroke. However, a recent study in a mouse model of Huntington's disease found that switching from glycolysis to oxidative fatty acid metabolism resulted in a neurotoxic astrocyte phenotype that enhanced ROS-induced damage [243]. The variability in the role of fatty acid metabolism in astrocytes may be due to varying pathological conditions, or it may be bidirectional.

Interestingly, reactive astrocytes release different lipids in response to different stimuli, leading to different consequences. For example, LPS-activated astrocytes selectively release PUFAs such as DHA [244], which reduces their proinflammatory responses. This may be one of the mechanisms by which astrocytes interact with the microglia. Moreover, saturated long-chain fatty acids activate inflammatory signaling in astrocytes [245], akin to microglia [154].

Although it remains unclear how fatty acid metabolism influences the phenotypic switching of reactive astrocytes in ischemic stroke, these novel findings suggest that astrocytic fatty acid metabolism may offer potential therapeutic avenues and new research opportunities for CI/RI (Table **1**).

## CONCLUDING REMARKS AND FUTURE PERSPECTIVES

Gliocytes are progressively being studied in ischemic stroke as supporting cells and key contributors to developing later CI/RI-related neuropathologies. In CI/RI, microglia and astrocytes undergo metabolic reprogramming to support their phenotypic changes and subsequent functional conversion due to stimulation by factors such as hypoxia, oxidative stress, and impaired energy metabolism. Maintaining proinflammatory functions within microglia requires elevated glycolysis, PPP, and fatty acid synthesis. Conversely, anti-inflammatory function requires heightened mitochondrial OXPHOS and fatty acid oxidative metabolism alongside specific fatty acid metabolites that act as secondary messengers of the cell, regulating microglial cell status. Despite extensive attempts to better understand the morphological and functional diversity of astrocytes during development and in ischemic stroke, the mechanisms regulating metabolic changes and reactive heterogeneity in astrocytes remain largely unknown. We could only establish that astrocyte glycolysis and glycogen and glutamate metabolism were strongly related to the pathological process of CI/RI. For CI/RI, therapeutic approaches targeting glial cells are more promising than those targeting neurons. We anticipate that therapeutic approaches that induce gliocyte phenotypic polarization and functional implementation by modulating their metabolic reprogramming will be an exciting therapeutic hotspot in the field of CI/RI. However, the dual nature of glial cells in ischemia necessitates a better understanding of the unique signaling pathways that trigger positive responses in each cell type.

As a new research hotspot in the field of CI/RI, several important issues require attention. First, precise molecular regulatory mechanisms governing metabolic reprogramming remain largely unknown. Although modern methods have been used to validate the glial cell morphological and functional diversity in higher mammalian species and rodents, it remains a challenge to determine the precise relationship between their morphological diversity and functional and metabolic diversity. Additionally, we suspect that other metabolic pathways, particularly iron metabolism, remain unexplored [38]. Second, since the pathophysiology of CI/RI is complicated and involves multiple factors, and microglial status and function are complex, it is important to regulate glial cell function and phenotype alone without precise disease stage knowledge. Different phenotypes may be crucial at specific disease stages, necessitating a deeper understanding of the pathogenic mechanisms underlying CI/RI. Finally, accurately regulating metabolic processes in glial cells remains challenging under current technical conditions, and most of the existing research has been performed in rodents, which may not be entirely typical of human glial cells *in vivo*. This review did not discuss oligodendrocytes, another important glial cell component. Since oligodendrocytes are relatively new to the field of cerebral ischemia, little is known about their function in this disease, requiring further attention. Despite these limitations, inducing gliocyte metabolic reprogramming may cause microglia and astrocytes to switch neurotoxic or neuroprotective phenotypes, potentially unlocking novel therapeutic avenues for CI/RI.

## Figures and Tables

**Table 1:** Metabolic therapeutic approaches *via* targeting microglia and astrocytes against CI/RI.

**Metabolic ** **Pathways**	**Drugs/Therapy**	**Target Diseases**	**Cell Types**	**Function or Effect**	**References**
Glucose	TIGAR	Hypoxic-ischemic brain damage	HAPI microglia	Elevating PPP flux/Decreasing ROS levels	[34]
	Lonidamine	Ischemia stroke	BV2 microglia	Inhibiting M1 activation	[71]
	CKLF1 inhibition	Ischemia stroke	Primary microglia	Promoting phagocytic activity	[72]
	Dichloroacetic acid and pyruvate	Ischemia stroke	Microglia *in vivo*	Inhibiting M1 activation	[74]
	Recombinant human CX3CL1	Ischemia stroke	Microglia *in vivo*/*vitro*	Promoting anti-inflammatory genes expression	[78]
	Salvianolic acid C	Cerebral ischemia reperfusion	BV2 microglia	Inhibiting cytokine release/ Inhibiting M1 activation	[80]
	STAT3 inhibition	Ischemia stroke	Primary astrocyte	Inhibiting glycolysis/ Inhibiting neurotoxicity	[19]
Glucose	TIGAR	Cerebral ischemia reperfusion	Primary astrocyte	Elevating PPP flux/ Inhibiting inflammation	[209]
	Dimethyl fumarate	Ischemia stroke	Primary astrocyte	Upregulate Nrf2 levels	[211]
Amino acids	Arginine	Ischemia stroke	Primary microglia/BV2 microglia	Inhibiting cytokine release	[116]
	Glycine	Ischemia stroke	Primary microglia	Inhibiting cytokine release/ Promoting M2 phenotype	[119]
	Glutaminase 1 inhibition	Ischemia stroke	Microglia *in vivo*	Inhibiting M1 activation/ Inhibiting exosome release	[124]
	Carnosine	Ischemia stroke	Primary astrocyte	Upregulate GLT-1 expression	[234]
	Ceftriaxone	Ischemia stroke	Primary astrocyte	Upregulate GLT-1 expression	[235]
	Tamoxifen	Ischemia stroke	Primary astrocyte	Upregulate GLT-1 expression	[238]
Fatty acids	DHA	Ischemia stroke	BV2 microglia	Inhibiting cytokine release	[171]
	PUFA n3	Ischemia stroke	BV2 microglia	Inhibiting pro-inflammatory genes expression	[172]
	PUFA n3	Ischemia stroke	Primary astrocyte	Inhibiting pro-inflammatory genes expression	[172]
	3,3,5 triiodo-L-thyronine	Ischemia stroke	Primary astrocyte	Elevating fatty acid oxidation/ Promoting neuroprotection	[242]
Glycogen	Insulin	Cerebral ischemia reperfusion	Primary astrocyte	Upregulate glycogen phosphorylase expression	[20]
	Salvianolic acid B	Cerebral ischemia reperfusion	Primary astrocyte	Enhancing glycogen phosphorylase activity	[201]

## LIST OF ABBREVIATIONS

A-FABP: Adipocyte Fatty Acid Binding Protein
Acetyl-CoA: Acetyl-coenzyme A
Acsl6: Long-chain acyl-CoA synthetase 6
AMPK: AMP-activated Protein Kinase
ApoE: Apolipoprotein E
Arg: Arginase
Aβ: Amyloid β-protein
BBB: Blood-brain Barrier
BCAA: Branched-chain Amino Acids
CD36: Cluster of Differentiation 36
CI/RI: Cerebral Ischemia/Reperfusion Injury
CKLF1: Chemokine-like Factor 1
CNS: Central Nervous System
DCA: Dichloroacetic Acid
DHA: Docosahexaenoic Acid
DKK: Dickkopf
EAAT: Excitatory Amino Acid Transport Proteins
EPA: Eicosapentaenoic Acid
ERK: Extracellular Regulatory Protein Kinases
F-2,6P: Fructose-2,6-bisphosphate
F6P: Fructose 6-phosphate
G6PD: Glucose-6-phosphate Dehydrogenase
GABA: Gamma-aminobutyric Acid
GLAST: Glutamate Aspartate Transporter Protein
GLT-1: Glutamate Transporter 1
GLUT1: Glucose Transporter Protein 1
GP: Glycogen Phosphorylase
GPR: G Protein-coupled Receptor
GS: Glutamine Synthetase
GSH: Reduced Glutathione
GSK3: PKA and Glycogen Synthase Kinase 3
HIF-1α: Hypoxia Inducible Factor-1α
HK: Hexokinase
HO-1: Heme Oxygenase-1
HSP72: Heat Shock Protein 72
IGF-1: Insulin-like Growth Factor-1
IL: Interleukin
iNOS: Inducible Nitric Oxide Synthase
IRG1: Immune Response Gene 1
JAK2: Janus Family Tyrosine Kinase 2
Keap1: Kelch-like ECH-associated Protein 1
LPL: Lipoprotein Lipase
LPS: Lipopolysaccharide
MAPK: Mitogen-activated Protein Kinase
MCAO: Middle Cerebral Artery Occlusion
MCT: Monocarboxylate Transporter
MMP: Matrix Metalloprotein
mTOR: Mammalian Target of Rapamycin
MUFAs: Monounsaturated Fatty Acids
NADPH: Triphosphopyridine Nucleotide
NHE1: Na/H Exchanger
NLRP3: TLR4-NOD-like Receptor Thermal Protein Domain Associated Protein 3
NO: Nitric Oxide
NOX: NADPH Oxidase
Nrf2: Nuclear Factor Erythroid 2-related Factor 2
OGD/R: Oxygen/Glucose Deprivation/Reoxygenation
OXPHOS: Oxidative Phosphorylation
PD: Pyruvate Dehydrogenase
PDK: Pyruvate Dehydrogenase Kinase
PEP: Phosphoenolpyruvate
PFK: Phosphofructokinase
PFKFB: 6-phosphofructo-2-kinase/fructose-2,6-bisphosphatase
PGC-1β: PPARγ-coactivator-1
PhK: PKA Glycogen Phosphorylase Kinase
PI3K: Phosphatidylinositol 3-kinase
PKM2: Pyruvate Kinase M2
PPP: Pentose Phosphate Pathway
PTEN: Tensin Homolog Deleted on Chromosome 10
PUFAs: Polyunsaturated Fatty Acids
RNS: Reactive Nitrogen Species
ROS: Reactive Oxygen Species
rtPA: Recombinant Tissue Plasminogen Activator
SDH: Succinate Dehydrogenase
SFAs: Saturated Fatty Acids
SNAT: Sodium-coupled Neutral Amino Acid Transporter
SOD2: Mitochondrial Superoxide Dismutase 2
SPMs: Specialized Pro-resolving Mediators
STAT: Signal Transducer and Activator Transcription
TCA: Tricarboxylic Acid
TIGAR: TP53-induced Glycolysis and Apoptosis Regulator
TLR4: Toll-like Receptor 4
TREM2: Triggering Receptor 2
